# Has the time come for doing away with Cobalt-60 teletherapy for cancer treatments

**DOI:** 10.4103/0971-6203.51931

**Published:** 2009

**Authors:** R. Ravichandran

**Affiliations:** Member, Editorial Board, Medical Physics Unit, Department of Radiotherapy, National Oncology Center, The Royal Hospital, Muscat, Oman. E-mail: ravichandranrama@rediffmail.com

Cancer disease tops among the causes of death, and in the year 2020 about 20 million new cancer cases may be expected globally. Majority of them will be in developing countries with more population, less financial resources, and other major priorities than health problems. More than 70% of all cancer deaths now occur in these countries and cancer kills more people every year than AIDS, malaria, and tuberculosis combined. Inter-Society Council for Radiation Oncology (ISCRO), USA[1] gives a direction that every patient with cancer deserves best possible management, either cure, long-term tumor control or palliation. World Cancer Report by the World Health Organization (WHO)[2] brought out the type of prevailing cancers in twelve world regions. It is seen that in most of the developing world with higher population, such as south central Asia, sub-Saharan Africa, cancers of cervix uteri, breast, oral cavity, forms the bulk of malignancies. The major goal for the future, therefore, should be to plan for providing treatment facilities for each cancer patient despite their stage at diagnosis. In these circumstances, clear strategies to address augmentation of infrastructure for cancer management is the need of the hour.

Radiotherapy (RT) is one of the major modalities of cancer treatment and about 60% of these patients require RT as curative or palliative intent. International guidelines^1^ recommend one megavoltage therapy equipment for every 1,20,000 population, for every 250 new patients providing about 6,250 treatments per year. In the above document, they have calculated based on the assumption that 50% of the patients could be treated for cure (30 to 40 increments) and 50% of the remaining, for palliation (10 to 20 increments). Therefore, 125 patients × 35 treatments (4375) and 125 patients × 15 treatments (1875), respectively, totaling to 6250 treatments. Taking all types of patients treated and various type of treatments, the above number appears legitimate for planning treatment facilities in an RT center. The equipment replacements must be justified based on departmental needs and not based on geographical or political need.[1]

At the outset, the scenario of radiation oncology infrastructure in most of the developing world remains discouraging, with only a handful of centers having modern facilities. Many centers still lack capabilities of simple localization iso-centric simulator x-ray machines, treatment planning systems, 3D imaging capabilities, and mould room facilities. The following argument clarifies above statement. For example, in India for a population of about 1,100 million, at the cancer incidence rate of 70 per 100,000 population, 60% of them requiring RT, we need about 1155 machines assuming a load of 400 per treatment machine annually. Presently, there are only 400 tele-therapy machines, about 25% of them served more than 10 years needing urgent replacements. Availability of less number of machines will likely to compromise correct patient care which will have implications in the optimal outcome. Therefore, the scenario needs improvement defenitely. However, it is heartening to note that recently in India, the rate of growth is about 25 machines per annum, which is a very good indicator of fast growth of radiotherapy infrastructure.

Also, in the recent past the trend is such that high technology centers cluster around corporate sector, unaffordable to common public, thereby increasing the gap between demand and supply. These centers alone cannot solve the total burden of cancer patients. It is also observed that, wherever replacements of existing tele-cobalt machines were taking place, they are being done with state-of-the-art linacs, which affect the total throughput number of treated patients in these institutions, leaving many patients not receiving treatments. Bulk of the patients are in low socio-economic group and therefore government hospitals and medical colleges only still have to offer services to these patients. In the existing scenario, there is a strong need to make policies to add more treatment machines in public funded institutions and improve the basic needs in these institutions, so that cancer care services are available to all sections of the society.

As early as 1980's, it was realized that a basic port film is definitely required to confirm reproducibility of treatment fields, and also execution of treatments with good immobilization techniques. Seldom have these techniques been implemented in most of the existing centers, which make it difficult for critical analysis of beam quality differences in the treatment outcomes. Without firm base and infrastructure, the clinical application of radiotherapy beams cannot produce optimal results.[3] A more optimistic statement may be that about 50% of existing scenario needs intervention and correction.

3D visualization methods and computed tomography (CT) imaging have become basic need for treatment planning both for localization and staging of disease. Basic localization, selection of beam center and field placement are possible with simple projected simulator radiograph, which also accounts for beam divergence simulating beam's eye view. If 3D delineated contour is not available, there is nothing better achieved with a multi-leaf collimator. In addition, if immobilization is not proper, a 3D treatment execution and beam direction cannot produce better treatment outcome in radiotherapy. In this context, for documentation of correct treatment plans, the role of a therapy simulator cannot be dispensed with. The ratio of sophisticated to simple treatments will be about 30:70 in the total number of patients and therefore it may be worthwhile to suggest one tele-cobalt machine for simple treatments and one low energy linac for conformal, 3D, intensity-modulated radiation therapy (IMRT) treatments.

In the above context, one looks for urgent solution. For large countries like India based on the incident spectrum of malignancies prevailing, World Health Organization (WHO)[4] recommended tele-cobalt machines as a simple effective equipment. As per the WHO, the main advantages of tele-cobalt machines should be given favorable consideration by the health administrators and governmental agencies. Another WHO report[5] on National Cancer Control Programmes, states that “relatively inexpensive cobalt machines are quite easy to maintain and can provide adequate therapy or palliation for most patients, thus making it unnecessary to invest in expensive linear accelerators and other high-energy machines requiring sophisticated maintenance and frequent calibration. For the majority of treatable cancers in developing countries, linear accelerators offer no advantage over cobalt therapy.” The physics factors of ^60^Co machines[6,7] versus high energy linacs need revisit by the clinical oncologists. Table 1 brings out the salient physical features of tele-cobalt beam compared to linac photon beams. ^60^Co machine is equivalent to a low energy linac of about 4 MV mean voltage, and provides an acceptable megavoltage photon beam for clinical applications. If the 5 mm build-up thickness is preserved by proper understanding of physics, there will be no problem of skin morbidities. Modern cobalt machines have penumbra trimmers, to cut down excess penumbra thereby reducing dose to critical structures adjacent to tumor volume. Radiation biology has not shown with statistical significance, differences in clinical outcome due to beam quality differences in general and there are no reports available in literature regarding lack of cure rates with simple treatments from ^60^Co machines. In addition, no one can deny the facts like simple infrastructure requirements (power supply, less power consumption, beam stability, and ease of operations) sufficient for tele-cobalt machines offering cost effective and un-interrupted treatments to large number of patients even in a rural set up where power fluctuations are commonly encountered.

**Table 1:** Comparison of various parameters for radiation sources of photon beams

*S. No*	*Physical and other Parameters*	*^60^Co machines*	*6 MV x-rays*	*15 MV/18 MV x-rays*
1	Build up	Equivalent to 4MV Build-up 5 mm	Emean ∼ 2 MeV Build up 15 mm	Emean ∼ 6 MeV Build up 28-35 mm
2	Skin dose	40-50%	∼ 25%	∼ 15-25%
3	Penumbra	90-10% is 1.5 cm Field definition 50%	Sharp beam field definition 80%	Sharp beam field definition 80%
4	Penetration	54% (10 cm)	67% (10 cm)	77% (10 cm)
5	Source distance	80 cm	100 cm	100 cm
6	Shape of isodose curves	Rounded beyond central zone (correctable)[9]	Flattened with special filter	Flattened with special filter
7	Side scatter	Less	Less	Less
8	Integral dose/tumor dose ratio	More for non-optimal plans. Manageable with good plans.	Less with simple fields	Less with simple fields
9	Absorption in bone	No differential absorption	No differential absorption	No differential absorption
10	Beam collimation	Asymmetric collimator (Yes)	Asymmetric collimators	Assymetric collimators
		MLC being tried	MLC, IMRT, SRT	MLC, IMRT
11	Irregular fields	Achievable with blocks. MLC being tried[10].	MLC, mMLC	MLC
12	Patients Registration	Possible	Networking yes	Networking yes
13	Computerized control console	Yes	Yes	Yes
14	Clinical acceptability	Yes (WHO,[2,4]	Yes	Yes
		Van Dyk,[6] Podgarsak,[7])		
15	Provision of performing tomotherapy	Feasibility reported'[11]	Dedicated machine	----

Therefore, with judicious treatment planning and intelligent executions of treatments, proper results could be achieved with tele-cobalt machines if basic facilities such as simulator and mould room are available. Reddy[8] made an analysis and indicated that low energy linacs (6 MV) are preferred to tele-cobalt machines, the main argument was related to cost of replacement of ^60^Co sources and management of decayed sources. Experience in the past has shown that replacements of magnetrons/klystrons also should be considered in linacs, along with increased maintenance costs. Management of decayed sources is possible by planning cascade loadings to a similar machine, so that higher dose rates with one machine could be possible, at the same time the life of the source could be prolonged. Attempts to obtain flattened isodose curves from telecobalt machines,[9] adopting multi-leaf collimators,[10] execution of tomotherapy with telecobalt machines[11] have added improvement of beam applications regarding the science of ^60^Co tele-therapy.

India has the technology for state-of-the-art tele-cobalt machines,[12] and possibility ^60^Co tele-therapy sources with output as high as 170 RMM (10000 RHM).[13] Dinshaw[14] advocated the need to revisit the context of cost effectiveness, cost benefit, and cost-utility analysis in Indian perspective and to strike the right balance between the science of technology and the art of medicine, with special relevance to radiotherapy in cancer treatments. The above statement is true to any developing country. All the above objective facts direct us to continue of use the well tested tele-cobalt beams for simple treatments to achieve cost-effective cure and palliation in cancer management. In addition, a low energy linac could be used for conformal 3D, IMRT treatments and should be available at each center. Therefore, it appears that the time has not come to do away with the tele-cobalt units.
